# Delay in Tuberculosis case detection in Pwani region, Tanzania. a cross sectional study

**DOI:** 10.1186/1472-6963-9-196

**Published:** 2009-10-29

**Authors:** Esther S Ngadaya, Godfrey S Mfinanga, Eliud R Wandwalo, Odd Morkve

**Affiliations:** 1 Centre for International Health, University of Bergen, Bergen, Norway; 2 Muhimbili Medical Research Centre, National Institute for Medical Research, Dar es Salaam, Tanzania; 3 Ministry of Health and Social Welfare, National Tuberculosis and Leprosy Control Programme, (NTLP), P.O. Box 9083 Dar es Salaam, Tanzania; 4 Management Sciences for Health, Dar es Salaam, Tanzania

## Abstract

**Background:**

Delay in Tuberculosis (TB) case detection may worsen the disease and increase TB transmission. It is also a challenge to the National TB and Leprosy control Program (NTLP).

**Methods:**

We conducted a cross sectional study in four out of six districts in Pwani region to estimate the extent and factors responsible for delay in TB case detection in Pwani region. Delays were divided into patient, health facility and total delay.

**Results:**

We enrolled a total of 226 smear positive TB patients. Out of 226 patient's results were available for 206. The majority (66.5%) of the patients were males. Mean age for males and females were 37.3 and 33.7 years respectively. Mean (SD) total delay was 125.5 (98.5) days (median 90). Out of 206 patients, 79 (38.35%) delayed to seek TB health care. Health facility delay was observed among 121 (58.7%) patients.

Risk factors for delay was poor knowledge that chest pain may be a TB symptom (OR = 2.9; 95%CI 1.20- 7.03) and the belief that TB is always associated with HIV/AIDS (OR = 2.7; 95%CI 1.39-5.23). Risk for delay was low among patients who first presented to a government health facility (OR = 0.3; 95%CI 0.12- 0.71) and those presenting with chest pain (OR = 0.2; 95%CI 0.10-0.61).

**Conclusion:**

There is a considerable delay in TB case detection in Pwani mainly contributed by patients. Risk factors for delay include misconception about TB/HIV and poor knowledge of TB symptoms.

## Background

Annually, about 2600 Tanzanians die from TB, which continues to be one of the major public health problems. The increased burden of TB in Tanzania is being fueled by HIV/AIDS [1].

A case of untreated smear positive tuberculosis can infect up to 15 people annually and over 20 during the natural course of untreated disease [2,3]. Early case detection and prompt treatment of infectious TB cases is the basis for achieving the millennium development goals, which aim to have halted and begun to reverse the incidence of TB by year 2015 [4].

TB case detection in Tanzania is mainly through passive case finding where patients present themselves to the health facility to seek care. However passive case finding depends much on the patient motivation and knowledge, financial capability, degree of suspiciousness of health workers, and the accuracy and effectiveness of diagnostic services [5]. Studies in Nigeria showed that 83% of patients presented in health facilities after a month or more from the onset of their symptoms [6]. In Ethiopia, the median patient and health facility delay were 60 and 6 days, respectively [7]. WHO estimates show that Tanzanian case detection rate is less than 50% [8]. Studies conducted in Tanzania and Botswana showed that patient from rural areas, patients with low education level, site of first visit, lack of TB information and female gender were associated with TB delay [9-12].

Except for the study conducted in two high TB burden cities of Mwanza and Dar es Salaam [8,9,11], the magnitude and factors responsible for delay in low TB burden regions of Tanzania is unknown.

This study was therefore, conducted to estimate the extent and factors responsible for delay in TB case detection in Pwani.

## Methods

### Setting

We conducted the study in Pwani region which is located in the eastern part of Tanzania Mainland (Coordinates 7°00'S, 39°00'E). The total population of Pwani in 2002 was 889,154 with 440,161 males and 448,993 females [13]. The study was conducted in four out of six districts located in Pwani region (Bagamoyo, Kibaha, Kisarawe and Mkuranga). Almost 73% of the population stays in the four districts studied (Bagamoyo 230,164; Kibaha132, 045; Kisarawe 95,614 and Mkuranga187, 428) [13]. Like in other parts of the country, TB services are free in all government facilities and health facilities are fairly well distributed with 90% of the population being within 10 kilometers from a health facility [8,14].

### Study design and data collection

We conducted a cross sectional hospital based study between April and October 2007. Four districts were randomly selected out of six districts in Pwani region. All four district hospitals were included into the study plus a random sample of 10% of all health facilities which offer TB services. In total we included the four hospitals, four health centers and eight dispensaries. All smear positive TB patients aged 15 years and above who were diagnosed within three months prior to the day of interview were enrolled and interviewed using a structured questionnaire which included open and close-ended questions. To ensure that all smear positive patients are enrolled, we identified all smear positive patients who have been diagnosed three months prior to the day of interview using registers before commencing data collection activities. We also enrolled smear positive patients who have just been diagnosed when the interview was going on. A maximum of two weeks was used to collect information in one facility depending on the number of smear positive patients available in the facility as well as patients drugs collecting schedule. We collected the following information: socio-demographic characteristics, knowledge about TB, place of first consultation and time spent to go to the nearest health facility. Other information collected were date of onset of pulmonary symptoms, date of first visit to a health facility, dates of collection of all three sputum samples, and date of starting treatment. If a patient did not remember the exact dates, he/she was asked if it was at the beginning of the month, at mid month or at end of the month. The beginning of the month was labeled as 5^th^, mid month was labeled as 15^th ^and end of the month was labeled as 25^th ^of the respective month. Patient TB treatment cards were also used to look at the date treatment was started.

We were granted ethical clearance to conduct this study by the Tanzania Medical Research Coordinating Committee which is the ethics coordinating body. We obtained informed verbal consent from each interviewee before enrolment. Data collectors were trained and questionnaires translated in Swahili and pre tested.

Standard procedure for the diagnosis of pulmonary tuberculosis in Tanzania is that all patients with cough of two or more weeks should collect three sputum samples in the form of "spot-morning-spot". Spot specimens are collected on the day the patient is suspected to have tuberculosis, morning samples are collected early in the morning of the second day and the third specimen is collected on submission of the morning specimen. Results of the sputum sample examinations should be communicated to the patient and treatment initiated on the same day after submission of the morning and spot specimens [1].

We calculated the sample size using Epi info version 6 on the assumption that the previous estimate of patient delay of more than 30 days for smear positive patients was 85% [9], total population of Pwani to be 900,000 and worst acceptable margin of 80% [15].

### Analysis

Data were double entered and cleaned using Epi data and analyzed using SPSS 11.5 for windows (SPSS Inc, Chicago, IL, USA). Description of each variable by delay was done. Risk factors for delay were estimated by bivariate logistic regression using cross tabulation with 95% confidence intervals (CI) given for odds ratios (OR) indicating statistically significant relationship if both values were above or below 1. Mean and median days of delay were calculated. We used the following time intervals:

**Patient delay**: the time interval between the day of experiencing for the first time one of the current pulmonary symptoms to the day the patient sought medical advice for the first time. Interval that exceeded 30 days was considered as patient delay [9,11]. **Health facility delay**: the time interval between first consultation at a health facility to the day the treatment was initiated. We considered a time interval of 5 days as health facility delay [11]. **Total delay**: the sum of the patient and health facility delay.

Patients who knew that TB can be spread from one person to another by coughing/sneezing were defined as having 'good' knowledge on TB transmission. Patients who mentioned prolonged cough plus two other symptoms from the following: fever, night sweat, chest pain, difficult in breathing, weight loss and coughing blood were defined as having good knowledge of TB symptoms [11].

## Results

### General patient's characteristics

We enrolled a total of 226 smear positive TB patients. The majority (66.5%) of the patients were males. Their mean (SD) and median age was 37.3 (14.5) and 35 years respectively. Mean (SD) and median age for females was 33.7 (12.8) and 31 years. Seventy nine patients (38.35%) delayed to seek TB health care for more than 30 days. Mean (SD) and median (range) time interval between onset of symptoms to first consultation at any health facility was 10.9 (9) and 9 (30) days respectively among patients who did not delayed to seek TB health care. Mean (SD) and median (range) patient delay among delayed patients was 75.8 (43.5) and 62 (181) days. Only 92 (44.7%) of the patients were suspected in their first visit. Fifty two (24.9%) patients were not started on treatment until more than three months from the onset of their illness. General patients' characteristics as risk factors for TB patient's diagnosis delay are shown in table 1. Patients who first presented to a government health facility had 0.3 (95%CI 0.12- 0.71) times the odds of delay compared to those who attended private health facilities.

**Table 1:** Socio-demographic characteristics as risk factors for patients delay.

	**Patient delay** **n (%)**	**No patient delay** **n (%)**	**Odds ratio and 95%CI**
Gender			
**Male**	58/79(73.42)	79/127 (62.20)	OR = 0.6 (95%CI 0.32-1.10)
**Female**	21/79(26.58)	48/127 (37.80)	
			
Marital Status			
**Single**	33/79 (41.77)	60/127 (47.24)	OR = 0.8(95%CI 0.45-1.41)
**Couple**	46/79 (58.23)	67/127 (52.76)	
			
Age group *			
**< 18 Years**	2/79(2.53)	6/124 (4.84)	
**18-40**	49/79(62.03)	80/124 (64.52)	OR = 0.5 (95%CI 0.10-2.80)
**> 40**	28/79(35.44)	38/124 (30.65)	OR = 05 (95%CI 0.08-2.41)
			
Education Level			
**No formal education**	33/79 (41.77)	60/127 (47.24)	OR = 0.8(95%CI 0.45-1.41)
**Completed primary school and above**	46/79 (58.23)	67/127 (52.76)	
			
Place of first presentation**			
**Government Facility**	47/78(60.26)	97/126 (76.98)	**OR = 0.3; (95%CI 0.12- 0.71)‡**
**Private facility**	16/78(20.51)	20/126 (15.87)	OR = 0.5; (95%CI 0.17- 1.38)
**Traditional Healers**	15/78(19.23)	9/126 (7.14)	
			
Time spent to go to the nearest Health facility***			
**30 minutes or less**	37/79 (46.8)	58/125 (46.4)	OR = 1.0 (95%CI 0.56-1.73)
**More than 30 minutes**	42/79 (53.2)	67/125 (53.6)	
			
HIV self reported****			
**HIV positive**	14/65 (21.5)	36/103 (35.0)	OR = 2.0 (95%CI 0.96- 4.01)
**HIV negative**	51/65 (78.5)	67/103 (65.0)	

*n = 3 were missing age,

** n = 2 were missing place of first consultation

***n = 2 missing time spent to go to the nearest facility

**** n = 38 were missing HIV status

### Presenting symptoms

The majority of the patients presented with a combination of symptoms. However, the most frequently reported symptoms were prolonged cough 78.6% (95%CI 73.00-84.2), evening fever 53.3% (95%CI 46.49-60.11), chest tightness (30.1%) (95%CI 23.84-36.36), weight loss 19.4% (95%CI 14.00-24.8), chest pain 18.5% (95%CI 13.20-23.80) and hemoptysis 13.1% (95%CI 8.49-17.71).

### Patient's knowledge on TB

Generally, 67 (32.5%) (95%CI 26.1-38.9) and 185 (89.8%) (95%CI 85.67-93.93) of patients had good knowledge on TB symptoms and possible ways of TB transmission, respectively. One hundred and seventy three patients (84.0%) (95%CI 78.99-89.01) were aware that prolonged cough is a TB symptom. Almost all patients (98.1%) (95%CI 96.24-99.96) were aware that TB is curable. Other symptoms mentioned were; evening fever (60.2%) (95%CI 53.53-66.88), difficulty in breathing (29.1%) (95%CI 22.9-35.3), loss of weight (20.9%) (95%CI 15.35-26.45), coughing blood (19.4%) (95%CI 14-24.8) and chest pain (17.0%) (95%CI 11.87-22.13).

### Risk factors for TB patients delay

Table 2 illustrates risk factors for patients delay. Patients who presented with chest pain were 0.2 times (95%CI 0.10-0.61) less likely to delay compared to those with no chest pain. Other risk factors associated with patients delay was a belief that TB is always associated with HIV/AIDS (OR = 2.7; 95%CI 1.39-5.23) and having poor knowledge that chest pain may be a TB symptom (OR = 2.9; 95%CI 1.20- 7.03).

**Table 2:** Risk factors for patients delay.

	**Delay** **n (%)**	**No delay** **N (%)**	**Odds ratio and 95%CI**
Presenting symptoms			
**Cough > 2 weeks**	64/79(81.0)	98/127(77.2)	OR = 0.8(95%CI 0.39-1.59)
**Cough with blood**	12/79(15.2)	15/127(11.8)	OR = 0.7(95%CI 0.33-1.69)
**Difficult in breathing**	23/79(29.1)	39/127(30.7)	OR = 1.1(95%CI 0.58-2.00)
**Chest Pain**	6/79 (7.6)	32/127(25.2)	**OR = 0.2(95%CI 0.10-0.61)‡**
**Fever**	44/79(55.7)	70/127(55.1)	OR = 1.0 (95%CI 0.56-1.72)
**Loss of weight**	20/79(25.3)	20/127(15.8)	OR = 0.6(95%CI 0.27-1.11)
			
Poor knowledge of TB symptoms			
**Cough > 2 weeks**	16/79(20.3)	17/127(13.4)	OR = 1.64(95%CI 0.78-3.48)
**Cough with blood**	58/79(73.4)	108/127(85.0)	OR = 0.5(95%CI 0.24-0.98)
**Difficult in breathing**	56/79(70.9)	90/127(70.9)	OR = 1.0(95%CI 0.54-1.86)
**Chest Pain**	72/79(91.1)	99/127(78.0)	**OR = 2.9(95%CI 1.20- 7.03)‡**
**Fever**	33/79(41.8)	49/127(38.6)	OR = 1.1(95%CI 0.64-2.02)
**Loss of weight**	60/79(75.9)	103/127(81.1)	OR = 0.7(95%CI 0.37-1.45)
			
Poor knowledge of transmission			
**Cough/Sneezing**	10/79(12.7)	11/127(8.7)	OR = 1.5(95%CI 0.62-3.78)
**Sharing eating utensils**	71/79(89.9)	109/127(85.8)	OR = 1.5(95%CI 0.60-3.55)
**Shaking hands**	31/79(39.2)	46/127(36.2)	OR = 1.1(95%CI 0.64-2.03)
**Mosquito bite**	47/78 (60.3)	64/127(50.4)	OR = 1.5(95%CI 0.84-2.64)
**Mother to child transmission during pregnancy**	66/79(83.5)	103/127(81.1)	OR = 1.2(95%CI 0.56-2.49)
			
Believe that TB is always associated with HIV/AIDS*	52/69 (75.4)	59/111 (53.2)	**OR = 2.7(95%CI 1.39- 5.23)‡**
			
Poor knowledge of TB curable	1/79(1.3)	3/127(2.4)	OR = 0.5(95%CI 0.05-5.19)

*n = 26 were missing

### Factors related to Patients and health facility delay among smear positive patients

Table 3 summarizes factors related to patients and health facility delay. There was no statistically significant difference when comparing factors associated with patients as well as health facility delay across gender, education level, presenting symptoms and knowledge of TB symptoms. This could mean that both patient and facility delays are impacting on TB problem equally.

**Table 3:** Factors related to events of patients and health facility delay among smear positive patients.

	**Patient delay** **n (%)**	**Health facility delay** **n (%)**	**No patient delay** **n (%)**	**No health facility delay n (%)**
Gender				
**Male**	58/79(73.42)	78/121 (64.5)	79/127(62.20)	59/85 (69.4)
**Female**	21/79(26.58)	43/121 (35.5)	48/127(37.80)	26/85 (30.6)
				
Marital Status				
**Single**	33/79(41.77)	58/121 (47.9)	60/127(47.24)	35/85 (41.2)
**Couple**	46/79(58.23)	63/121 (52.1)	67/127(52.76)	50/85 (58.8)
				
Age group				
**< 18 Years**	2/79(2.53)	6/119 (5.0)	6/124 (4.84)	1/84 (1.2)
**18-40**	49/79(62.03)	73/119 (61.3)	80/124(64.52)	57/84 (67.8)
**> 40**	28/79(35.44)	40/119 (33.6)	38/124(30.65)	26/84 (31.0)
				
Education Level				
**No formal education**	33/79(41.77)	56/121 (46.3)	60/127(47.24)	37/85 (43.5)
**Completed primary school and above**	46/79(58.23)	65/121 (53.7)	67/127(52.76)	48/85 (56.4)
				
HIV self reported				
**HIV positive**	14/65(21.5)	29/101 (28.7)	36/103 (35.0)	21/68 (30.9)
**HIV negative**	51/65(78.5)	72/101 (71.3)	67/103 (65.0)	47/68 (69.1)
Presenting symptoms				
**Cough > 2 weeks**	64/79(81.0)	93/121 (76.9)	98/127(77.2)	68/85 (80.0)
**Cough with blood**	12/79(15.2)	14/121 (11.6)	15/127(11.8)	13/85 (15.9)
**Difficult in breathing**	23/79(29.1)	36/121(29.8)	39/127(30.7)	27/85(31.8)
**Chest Pain**	6/79(7.6)	29/121(24.0)	32/127(25.2)	10/85(11.8)
**Fever**	44/79(55.7)	65/121(53.7)	70/127(55.1)	50/85(58.8)
**Loss of weight**	20/79(25.3)	19/121(15.7)	20/127(15.8)	21/85(24.7)
Poor knowledge of TB symptoms				
**Cough > 2 weeks**	16/79(20.3)	19/121(15.7)	17/127(13.4)	14/85(16.5)
**Cough with blood**	58/79(73.4)	102/121(84.3)	108/127(85.0)	64/85(75.3)
**Difficult in breathing**	56/79(70.9)	83/121(68.6)	90/127(70.9)	63/85(74.1)
**Chest Pain**	72/79(91.1)	98/121(81.0)	99/127(78.0)	73/85(85.9)
**Fever**	33/79(41.8)	47/121(38.8)	49/127(38.6)	34/85(40.0)
**Loss of weight**	60/79(75.9)	97/121(80.2)	103/127(81.1)	65/85(76.5)

### Health facility delay

Health facility delay was observed among 121 (58.7%; 95%CI 51.98-65.42) patients. Of these, 78 (64.5%; 95%CI 57.97-71.03) were males and 43 (35.5%: 95%CI 28.97-42.03) were females. Mean (SD) and median (range) health facility delay was 49.7 (56.0) and 28.0 (262) days. Seventy three (61.3%; 95%CI 54.65-67.95) were between 18-40 years. The majority 65 (53.7%; 95%CI 46.89-60.51) completed primary school (table 4). Mean (SD) and median (range) time interval between first consultation to any health facility and initiation of treatment was 2.3 (1.4) and 2.0 (5.0) days respectively among patients with no health facility delay.

**Table 4:** Risk factors for health facility delay

	**Health facility delay n (%)**	**No health facility delay n (%)**	**Odds ratio and 95%CI**
Gender			
**Male**	78/121 (64.5)	59/85 (69.4)	OR = 0.8(95%CI 0.44-1.44)
**Female**	43/121 (35.5)	26/85 (30.6)	
			
Marital status			
**Single**	58/121 (47.9)	35/85 (41.2)	OR = 0.8(95%CI 0.43-1.33)
**Couple**	63/121 (52.1)	50/85 (58.8)	
			
Age group *			
**< 18 Years**	6/119 (5.0)	1/84 (1.2)	
**18-40**	73/119 (61.3)	57/84 (67.8)	OR = 0.3(95%CI 0.03-2.25)
**> 40**	40/119 (33.6)	26/84 (31.0)	OR = 0.2(95%CI 0.02-1.82)
			
Education level			
**No formal education**	56/121 (46.3)	37/85 (43.5)	OR = 0.9(95%CI 0.51-1.56)
**Completed primary school and above**	65/121 (53.7)	48/85 (56.4)	
			
HIV self reported**			
**HIV positive**	29/101 (28.7)	21/68 (30.9)	OR = 0.9(95%CI 0.46-1.76)
**HIV negative**	72/101 (71.3)	47/68 (69.1)	
			
Presenting symptoms			
**Cough > 2 weeks**	93/121 (76.9)	68/85 (80.0)	OR = 0.8(95%CI 0.42-1.64)
**Cough with blood**	14/121 (11.6)	13/85 (15.9)	OR = 0.7(95%CI 0.32-1.63)
**Difficult in breathing**	36/121(29.8)	27/85(31.8)	OR = 0.9(95%CI 0.50-1.66)
**Chest Pain**	29/121(24.0)	10/85(11.8)	OR = 2.4(95%CI 1.08-5.16)
**Fever**	65/121(53.7)	50/85(58.8)	OR = 1.2(95%CI 0.70-2.16)
**Loss of weight**	19/121(15.7)	21/85(24.7)	OR = 1.2(95%CI 0.64-2.43)
			
Poor knowledge of TB symptoms			
**Cough > 2 weeks**	19/121(15.7)	14/85(16.5)	OR = 0.9(95%CI 0.44-2.00)
**Cough with blood**	102/121(84.3)	64/85(75.3)	OR = 1.8(95%CI 0.88-3.53)
**Difficult in breathing**	83/121(68.6)	63/85(74.1)	OR = 1.0(95%CI 0.62-1.58)
**Chest Pain**	98/121(81.0)	73/85(85.9)	OR = 1.3(95%CI 0.90-1.95)
**Fever**	47/121(38.8)	34/85(40.0)	OR = 1.0(95%CI 0.54-1.68)
**Loss of weight**	97/121(80.2)	65/85(76.5)	OR = 1.2(95%CI 0.64-2.43)

*n = 3 were missing age

** n = 37 were missing HIV status

### Total delay

Mean (SD) and median time interval between onset of symptoms to initiation of treatment was 125.5 (98.5) and 90.0 days respectively among patients who delayed to seek TB health care.

## Discussion

Our study indicates that 79 (38.4%) patients delayed to seek TB health care. Thirty days was considered as a cut off point for patient delay, taking into account the local situation of these communities and other studies conducted in Tanzania [9,11]. Cut off point for health facility delay was set at 5 days. The mean time interval between onset of symptoms to first consultation at any health facility was 75.8 days among patients who delayed to seek TB health care, and these patients may serve as potential reservoirs for infection.

The proportion of patients who delayed was not as high as what has been found in other studies [9,15], and is smaller than what was found in Mwanza [9]. However, it is almost the same as previously reported from Dar es Salaam [11]. Though not investigated in this study, the differences in delay could probably be explained by the study site, cultural factors and increased awareness of TB among communities since 2000 when the study in Mwanza was conducted.

Almost a quarter of patients were not started on treatment until more than three months from the onset of their illness. This is similar to what has been found in Ethiopia [7]. The major contributor to the total delay observed in this study was the delay of patients (63%) but this was lower than what was found in Mwanza where patient contributed to the total delay by more than 90% [9]. Studies in Ethiopia and Nigeria also show dominance of patients delay in the total delay [6,7]. Patients take long time before diagnosis when considering both patient and health system delay of more than 35 days. This has implication on delayed case detection hence increased transmission in communities since TB patients would have stayed longer in the community before diagnosis and treatment. Public interventions are therefore inevitable if we are to reduce TB transmission in the community and increase case detection rate. Interventions targeting change of health seeking behavior, ways of increasing diagnostic suspicion index of health personnel and improving laboratory methods would reverse the transmission trends.

Patients with symptom of chest pain and those who first presented to government health facilities were less likely to delay to seek TB health care. This may be partly related to TB services which are mostly offered in government compared to private facilities because TB services are free of charge. Interventions to improve early case detection and treatment should also target TB service in private facilities, and we thus recommend to put more effort to improve public private partnership in TB control in the country.

In addition, patients with poor knowledge that chest pain was one of the TB symptoms and those who believe that TB is always associated with HIV/AIDS delayed to seek TB health care. This finding is similar to a study conducted in Dar es Salaam [11]. Though not investigated in this study, similarities of some of TB symptoms with that of HIV/AIDS and stigma associated with HIV/AIDS could offer an explanation.

Level of education attained and gender had no significant effect on delay of seeking TB health care, similar to findings in Uganda [15]. However, this is in contrary to studies conducted in Dar es Salaam and Mwanza [9,11]. Furthermore, patients delay was not significantly associated with self reported HIV/AIDS status. Though we did not investigate whether these patients were tested before or after TB diagnosis, it is well known among TB health workers that every TB patient should be HIV tested [1]. Therefore, if they had HIV test following TB diagnosis, their HIV/AIDS status would not affect their health seeking behavior.

Likewise, health care seeking observed in this study differs from other studies. More patients in our study first sought help for their pulmonary symptoms in government hospitals, in contrast to a study in India, which showed a high proportion of TB patients first seeking health care in private facilities [16]. However, despite that more patients in our study first sought health care for their pulmonary symptoms in government facilities, yet more than 55% delayed to be suspected at their first visit, even if many of them (78.6%) had prolonged cough of more than two weeks prior to their first consultation. Although our study did not assess the availability of TB diagnostic services in the facilities where patients visited for their first consultation but the NTLP guidelines requires clinicians working in facilities with no TB diagnostic services to refer patients or send patient's sputum early to a facility with TB diagnosis services for TB investigation [1]. Unfortunately, in most cases the guidelines are not always well known and are even less well followed by health care providers. How much the guidelines are known and followed is an area which needs further studies.

Other limitations of the study include recall bias on estimation of delay. Our data analysis did not use random effect model to adjust for possible individual or practice variations. The data was not sufficient enough to use the model. This could have some effect in the 95% Confidence Interval. However, in longitudinal and cluster trial studies involving repeated measure of parameter estimates this bias can lead to invalid inferences regarding measures of effect such as risk ratios (RR) or OR [17].

## Conclusion

There is a considerable delay in TB case detection in Pwani mainly contributed by patients. Risk factors for delay include misconception about TB/HIV and poor knowledge of TB symptoms. Interventions are required to change public health seeking behavior so as to reduce patient delay, and to equip and train health personnel at facility level so as to eliminate health system delay.

## Competing interests

The authors declare that they have no competing interests.

## Authors' contributions

ESN is the primary author who was responsible for conceiving of the research idea, designing of the study, collection of data, analysis and interpretation of the results and writing of the draft and final manuscript. She is also the corresponding author. GSM, ERW and OM participated in proposal write up, data analysis and interpretation of the results, and writing of the draft and final manuscript.

## Pre-publication history

The pre-publication history for this paper can be accessed here:


